# Iron-Nickel Alloy with Starfish-like Shape and Its Unique Magnetic Properties: Effect of Reaction Volume and Metal Concentration on the Synthesized Alloy

**DOI:** 10.3390/nano11113034

**Published:** 2021-11-12

**Authors:** Norhan Nady, Noha Salem, Marwa A. A. Mohamed, Sherif H. Kandil

**Affiliations:** 1 Polymeric Materials Research Department, City of Scientific Research and Technological Applications (SRTA-City), New Borg El-Arab City, Alexandria 21934, Egypt; 2 Department of Materials Science, Institute of Graduate Studies and Research, Alexandria University, Alexandria 21526, Egypt; noha.salem@yahoo.com (N.S.); s.kandil@usa.net (S.H.K.); 3 Fabrication Technology Department, City of Scientific Research and Technological Applications (SRTA-City), Borg El-Arab City, Alexandria 21934, Egypt; marwa945@yahoo.com

**Keywords:** starfish-like shape, iron–nickel alloy, magnetic properties, chemical reduction method

## Abstract

Iron-nickel alloy is an example of bimetallic nanostructures magnetic alloy, which receives intensive and significant attention in recent years due to its desirable superior ferromagnetic and mechanical characteristics. In this work, a unique starfish-like shape of an iron-nickel alloy with unique magnetic properties was presented using a simple, effective, high purity, and low-cost chemical reduction. There is no report on the synthesis of such novel shape without complex precursors and/or surfactants that increase production costs and introduce impurities, so far. The synthesis of five magnetic iron-nickel alloys with varying iron to nickel molar ratios (10–50% Fe) was undertaken by simultaneously reducing Fe(II) and Ni(II) solution using hydrazine hydrate as a reducing agent in strong alkaline media for 15 min at 95–98 °C. The effect of reaction volume and total metal concentration on the properties of the synthesized alloys was studied. Alloy morphology, chemical composition, crystal structure, thermal stability, and magnetic properties of synthesized iron-nickel alloys were characterized by means of SEM, TEM, EDX, XRD, DSC and VSM. ImageJ software was used to calculate the size of the synthesized alloys. A deviation from Vegard’s law was recorded for iron molar ration higher than 30%., in which superstructure phase of FeNi_3_ was formed and the presence of defects in it, as well as the dimensional effects of nanocrystals. The saturation magnetization (Ms), coercivity (Hc), retentivity (Mr), and squareness are strongly affected by the molar ratio of iron and nickel and reaction volume as well as the total metal concentration.

## 1. Introduction

In recent years, nanomaterials have begun to revolutionize the world as they provide new dimensions for research and development. Nanoparticles (NPs) are ultrafine particles with lengths ranging from 1 to 100 nm at least one dimension. They have a high surface-area-to-volume ratio, which enables them to exhibit different physical and chemical properties such as reactivity, versatility, and strength than their bulk counterparts [1,2]. Among various nanoparticle types, magnetic nanoparticles (MNPs) attracted increased attention due to their unique physicochemical and magnetic properties [3,4]. MNPs are potentially employed in many applications such as biomedicine [2,5], environmental remediation [1,4], catalysis [6,7], magnetic resonance imaging [8,9], data storage [10,11], sensors [12,13], electromagnetic shielding [14], and absorbing materials [15], etc.

The hysteresis loop has been used for characterizing the magnetic materials. One of the important magnetic properties is coercivity (Hc), which is a measure of the ability of a ferromagnetic material to keep its magnetization (i.e., without being demagnetized) when exposed to an external magnetic field [16]. Ferromagnetic materials with high coercivity are called magnetically hard and are used to make permanent magnets [17]. Materials with low coercivity are known as soft magnetic materials, which are used in many applications such as transformers [18], recording heads [19], microwave devices [20], and magnetic shielding [21]. Another important magnetic parameter is the saturation magnetization (Ms), which is the maximum possible magnetization of ferromagnetic material, characterized by parallel orientation of all magnetic moments inside the material; it decides that the materials are hard or soft magnets. Remanence or Retentivity (Mr) is the remaining magnetization left after removing the material from a magnetic field. A hard magnetic material has high remanence and coercivity [22]. Another parameter used to describe the magnetic materials is receptivity (Hr), which shows the willingness of the material to be magnetized. The ratio Mr/Ms, which is also called squareness ratio, measures how the square is the hysteresis loop and is a dimensionless quantity between zero and one. Its value depends on the target application; for example, it should be near 1 for memory devices, whereas it should be near zero for magnetic fluids.

Iron-nickel alloy is an example of bimetallic nanostructures magnetic alloy, which received intensive and significant attention in recent years due to its desirable superior ferromagnetic and mechanical characteristics that make it applicable in many fields [23,24,25]. Fe_x_Ni_1−x_ alloy with x being in the range of 20–50% molar ratio is called Permalloy, known for its soft magnetization, and widely used for magnetic heads and environmental remediation [14]. An attractive feature of Fe-Ni alloy is that it can be prepared with different sizes, shapes, compositions, morphologies, and size distribution depending on the method used for the preparation [23,25,26] and iron-to-nickel composition in alloy [23]. A variety of morphological structures of iron-nickel alloy have been prepared including spherical particles [27], triangular particles [28], rods [29], wires [23], platelets [30], flower-like [31], and leaf-like particles [32].

Fabrication of iron-nickel alloyed micro-/nanoparticles has been achieved by various physical and chemical methods such as mechanical alloy [33], chemical reduction in solution [34], chemical vapor deposition [35], physical vapor deposition [36], sol-gel combined with hydrogen reduction [37], sol-gel combustion [38], vacuum evaporation [39], electroless plating [40], chemical reduction based on the polyol process [41], microemulsion method [42], hydrothermal reduction [43], etc. Most of these processes usually require complex precursors and/or surfactants, which increase production costs and introduce impurities, resulting in noticeable declines in the properties of the structures [44]. Thus, a simple and low-cost process for the large-scale preparation of pure nanocrystalline magnetic iron-based alloy remains challenging to researchers. Moreover, additional efforts are needed to control experimental conditions for the synthesis of magnetic nanoparticles with controlled shape, size, and magnetic properties.

The chemical reduction method has advantages over other methods including mildness, simplicity, low cost, and large-scale production [34], and has been adopted to successfully prepare some low-dimensional Fe-Ni alloy nanostructures including nanospheres [26] and polycrystalline nanorods [27]. However, the fabrication of Fe-Ni alloy nanostructures via a solution-phase chemical reduction approach still remains a challenge because the ferrous ion is easily oxidized iron oxides and tends to hydrolyze. By control of their properties through the manipulation of their physical and molecular structures, researchers have developed iron oxide nanoparticles to support a wide range of applications.

The starfish-like structure presented in this work is a novel Fe-Ni alloy microstructures/nanostructures and there is no report on the synthesis of it by chemical reduction method so far. This work documents the study of the effect of the volume of the reaction solution (preparation) on the purity and (magnetic) properties of Fe-Ni alloys, which represents the possibility of preparing the mixture on an industrial scale and in a very short preparation time without affecting the purity and properties of the resulting alloy. In this study, five nanostructured Fe-Ni alloys were synthesized with varying iron to nickel molar ratios (10–50% Fe). The effect of the molar ratio between iron and nickel and total metal concentration as well as the reaction volume in the microstructure and the magnetic properties of the produced alloys was studied. Different analyses techniques such as SEM, TEM, EDX, XRD, DSC were used to observe the morphology and verify the composition of the synthesized alloys. ImageJ software was used to calculate the size of the synthesized alloys. Starfish-like Fe_10_ Ni_90_ alloy, which has unique magnetic properties, was investigated and presented in this work.

## 2. Materials and Methods

### 2.1. Materials

The chemicals used for alloy synthesis include nickel chloride hexahydrate (NiCl_2_·6H_2_O, 98%), Ferrous chloride tetrahydrate (FeCl_2_·4H_2_O, 99.99%) as sources of metal ion was purchased from Sigma (Darmstadt, Germany). Hydrazine hydrate (N_2_H_4_·H_2_O, 99%) was obtained from Fisher (Horsham, UK) as a reducing agent. Sodium hydroxide (NaOH, 98%) catalyst was purchased from trading dynamic co. TDC (Cairo, Egypt). Distilled water was used as a solvent.

### 2.2. Methods

Nanocrystalline of Fe_10_ Ni_90_, Fe_20_ Ni_80_, Fe_30_ Ni_70_, Fe_40_ Ni_60_, and Fe_50_ Ni_50_ alloys were synthesized as follows: Aqueous solutions of Fe^+2^, Ni^+2^ ions of molar ratio 1:9, 2:8, 3:7, 4:6, and 5:5 were prepared by dissolving appropriate amounts of FeCl_2_·4H_2_O and NiCl_2_·6H_2_O in distilled water. The prepared solution was vigorously stirred on a magnetic stirrer equipped with a heating unit at 1400–1600 rpm and 95–98 °C. The second solution of warm aqueous hydrazine, N_2_H_4_·H_2_O, (99 wt.%), and aqueous NaOH (0.1 M) with pH 12.8 was added to the first solution. The volumetric ratio of N_2_H_4_·H_2_O/NaOH solutions was about 5:1. The molar ratio of hydrazine to metal ions was significantly higher than the stoichiometric one. The precipitation of fine black particles was the result of the reduction reaction. The final fabricated black particles were separated magnetically, then washed repeatedly with distilled water until neutral pH and dried in a vacuum oven at 35 °C for 24 h. The experiment for each alloy was performed twice—once at small reaction volume (40 mL of aqueous solution) and the second was undertaken using a larger scale (80 mL of aqueous solution). Two metal concentrations were used in this work: 0.1 and 0.3 M with large reaction volumes.

### 2.3. Characterization

#### 2.3.1. X-ray Diffraction (XRD) Analysis

X-ray diffraction (XRD) was employed to characterize the synthesized alloys and determine their crystallite size and lattice parameter. XRD measurements were carried out on a Shimadzu XRD-7000 diffractometer (Kyoto, Japan, 45 kV, 30 mA; CuKα + Ni-filtered radiation, λ = 0.15406 nm). The 2θ range was 5–80°, at a scanning rate of 4°/min and a scanning step of 0.026°.

#### 2.3.2. Scanning Electron Microscopy (SEM) Imaging and X-ray Spectroscopy (EDX) Analysis

The morphology and particle sizes were analyzed by scanning electron microscopy (SEM, JEOL, Model JSM 6360 LA, Kyoto, Japan). SEM samples were prepared by dispersion of alloy particles on a cupper-support using a double face. The dispersed particles on the cupper-support were coated with Au before imaging. A voltage of 15 kV and a resolution of 1280 × 960 pixels were used. Chemical compositions were estimated by an area analysis using energy-dispersive X-ray spectroscopy (EDX) system equipped with SEM. Particle size was calculated using SEM images by imageJ software.

#### 2.3.3. Scanning-Transmission Electron Microscope (STEM) Imaging and X-ray Spectroscopy (EDX) Analysis

The particle morphology of the prepared alloys was inspected by high-resolution TEM (HREM) using a field emission electron-source scanning-transmission electron microscope (STEM) (JEOL 2010 F, Boston, MA, USA), operated at 200 kV. For the TEM investigations, the nanoparticles were deposited on a cupper-grid-supported transparent carbon foil. The sample was vigorously sonicated in ethanol for 15 min before deposition on the grade. Chemical compositions were estimated by an area analysis using an energy-dispersive X-ray spectroscopy (EDX) system equipped with STEM. Particle size, as well as the length of cones/needles, were calculated using STEM images by ImageJ software for starfish-like monocrystalline alloy.

#### 2.3.4. Vibrating Sample Magnetometer (VSM) Analysis

A vibrating sample magnetometer (VSM, Lake Shore 7410, Boston, MA, USA) was used to measure the room-temperature magnetic properties of the prepared alloys. The applied field was −20 ≤ H ≤ 20 kOe. For magnetization measurements, the powder was pressed strongly and fixed in a small cylindrical plastic box.

#### 2.3.5. Differential Scanning Calorimeter (DSC) Analysis

The thermal properties of the synthesized nanopowders were determined with differential scanning calorimetry (TA-4000 System Mettler, Auckland, New Zealand). A powder sample (5 mg) was heated from room temperature (27 °C) to 50 °C under air nitrogen atmosphere at a rate of 10°/min.

## 3. Results and Discussion

**Figure 1:**
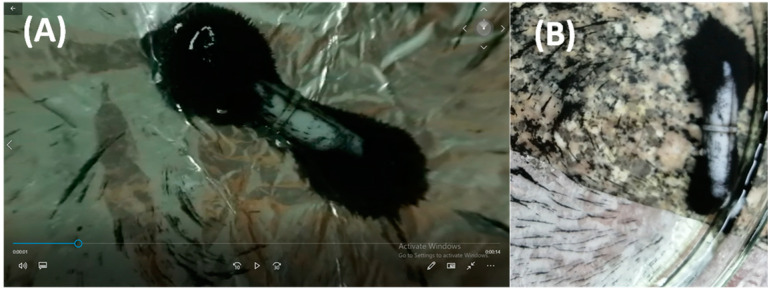
Photos of the prepared iron-nickel alloy during washing process (**A**,**B**).

Iron-nickel alloys have been given attention because of a broad variety of magnetic properties that can be obtained by manipulating the composition and the used preparation method. In this work, nanostructured iron-nickel alloys were synthesized within 15 min reaction time and their affinity to a magnet is high for collection with a magnet, during the washing process as shown in Figure 1.

### 3.1. Alloy Morphology

**Figure 2:**
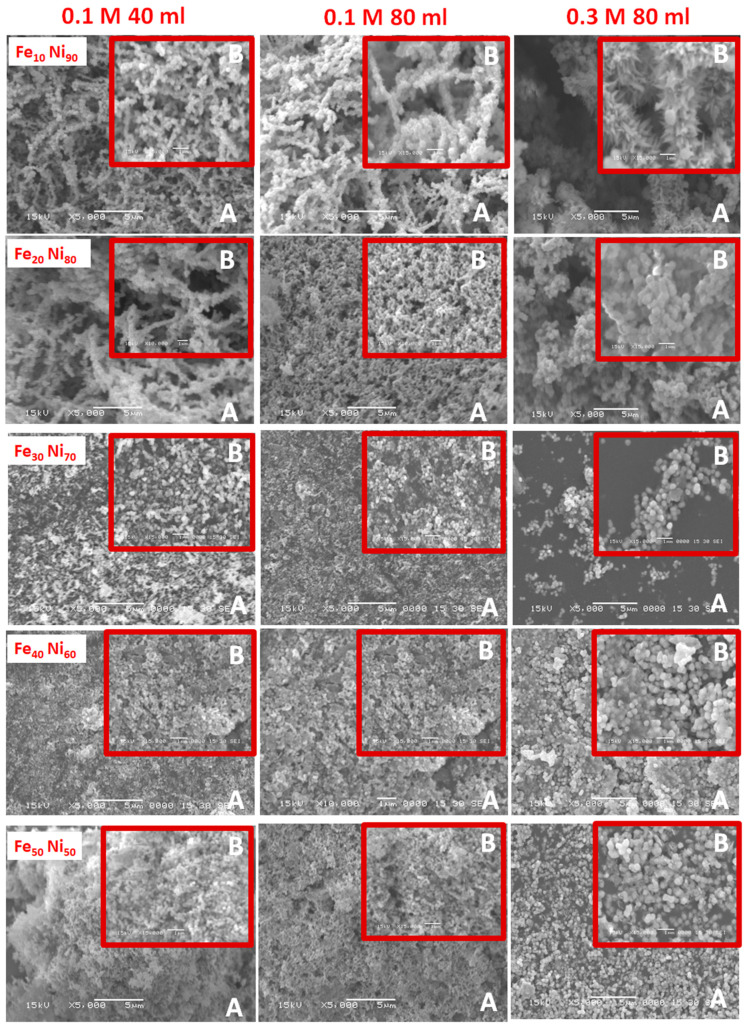
SEM images of the prepared iron-nickel alloy at two magnifications; (**A**) ×5000 and (**B**) ×15,000.

**Figure 3:**
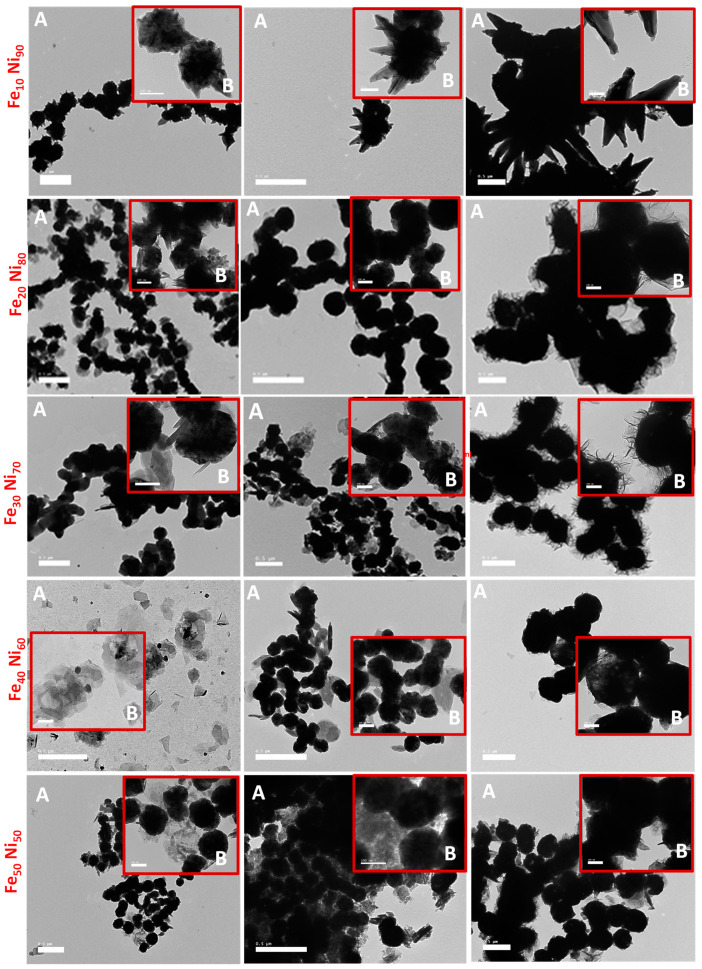
TEM images of the prepared iron-nickel alloy at two magnifications; (**A**) ×5000 and (**B**) ×15,000.

To obtain visual insight into the synthesized iron-nickel alloys, their morphologies were studied via the SEM and TEM techniques, shown in Figure 2 and Figure 3. The SEM images suggest that the nanoparticles of all the prepared alloys tend to agglomerate together when deposited onto the cupper-support, which can be attributed to the tendency of the alloy particles to decrease the system energy because of its high surface free energy and the magnetostatic energy. The molar ratio Fe_10_ Ni_90_ showed a completely different structured alloy than all the prepared alloys in which a unique starfish-like shape was captured. The clearer starfish-like shape with sides cones/needles was observed by SEM (Figure 2) with 0.3 M and 80 mL reaction volume. This morphology neither changed with doubling the reaction volume nor with increasing the metal concentration (40 and 80 mL reaction volumes and 0.1 and 0.3 M metal concentrations) as shown in both SEM (Figure 2) and TEM (Figure 3) images.

This starfish diminished with an increase in the iron content to Fe_20_ Ni_80_ molar ratio; however, a few cones/needles showed with small reaction volume (0.1 M with 40 mL reaction volume). Doubling the reaction volume and/or tripling the metal concentration resulted in replacing the cones/needles with hair around the formed necklace-like nanostructured alloy, especially with high metal concentration (0.3 M metal concentration with 80 mL reaction volume) as shown in TEM images (Figure 3). Increasing the molar ratio to Fe_30_ Ni_70_ also resulted in the necklace structure, but some clearer particles became predominate in the imaged alloy. Higher molar ratios (i.e., Fe_40_ Ni_60_ and Fe_50_ Ni_50_) resulted in diminishing the necklace-like nanostructured and formation of particle clusters, which may be attributed to the increase in the percentage of multi-domain particles.

**Figure 4:**
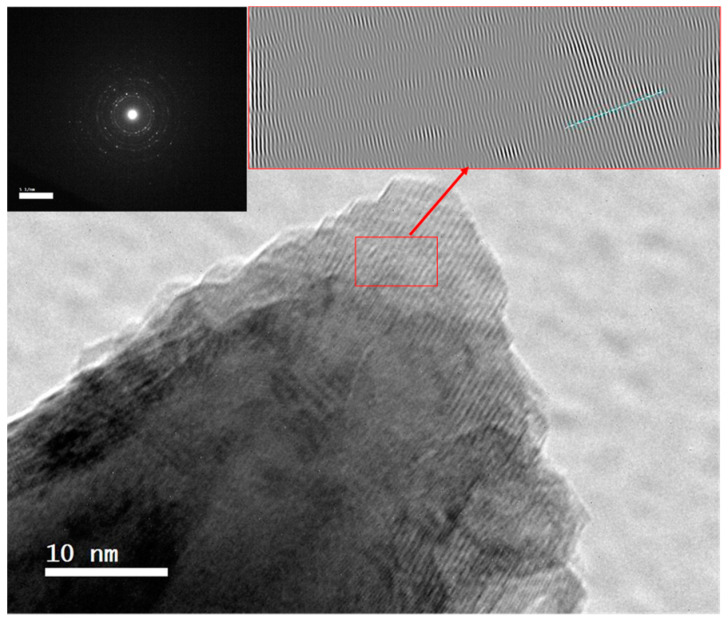
The high resolution TEM images of Fe_10_ Ni_90_ molar ratio synthesized using 0.1 M metal concentration and 80 mL reaction volume carried out for 15 min at 1400 rpm and 95–98 °C.

The high-resolution TEM image shown in Figure 4 of the selected area in the sides cones shows a good crystalline character with a lattice spacing of 0.207 nm; this can be indexed to the (111) plane of face-centered cubic FeNi nanocrystals, which suggested the cones grow along the (111) direction in which the magnetic particles grow along the magnetic easy axis of (111) direction.

### 3.2. Alloy Particle Size

**Figure 5:**
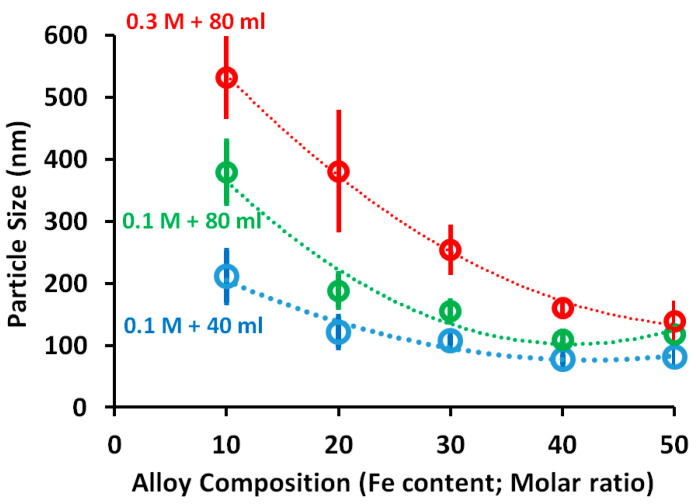
Particle size as function of the molar ratio of iron using ImageJ software; 40 mL reaction volume (blue circle) and 80 mL reaction volume (green circle) with 0.1 M metal concentration, 80 mL reaction volume with 0.3 M concentration (red circle).

The particle size of the syntheses’ alloys was calculated using ImageJ software for SEM and TEM images and the obtained results are presented in Figure 5. The starfish-like shape image has a size of 211 ± 42 nm for 0.1 M metal concentration with 40 mL reaction volume. The average size of the alloy increased by around 80% to 379 ± 50 nm with doubling the volume of the reaction solution (0.1 M metal concentration with 80 mL reaction volume). Also, an increase of around 152% occurred with the metal concentration being increased threefold (0.3 M metal concentration with 80 mL reaction volume) to reach 532 ± 53 nm. The images were also interpreted and confirmed by calculating the particle size through ImageJ software for the images of TEM. The TEM images illustrated that the reason for this growth of the particle size with an increase in either the reaction volume and/or metal concentration is due to the growth of the cones/needles in length by approximately 220 and 328% longitudinally and almost by 42 and 212% crosswise with doubling the reaction volume and tripling the total metal concentration, respectively. The effect of cones/needles on the measured particle size increased by around 20% (211 and 169 nm particle size with and without cones/needles, respectively) for 0.3 M metal concentration with 80 mL reaction volume to around 50% by doubling the reaction volume (379 and 246 nm particle size with and without cones/needles, respectively) or/and tripling the total metal concentration (532 and 300 nm particle size with and without cones/needles, respectively).

On the other hand, the calculated particle size decreased with increases in the iron molar ratio, which can be related to changing the particle shapes from starfish-like through the necklace-like structures to clear particles. However, a few cones/needles showed with small reaction volume (0.1 M with 40 mL reaction volume) at 20% iron molar ratio (Fe_20_ Ni_80_) resulted in an increase in the gap between the calculated sizes, which increase the standard deviation as shown in Figure 4 at this point. In general, the effect of an increase in the total metal concentration on the particle size of the alloys is more visible than the effect of doubling the reaction volume at the same total metal concentration (0.1 M).

### 3.3. Alloy Composition

Keeping in mind that EDX is not a tool for precision chemical analysis, (it is just an instrument for estimation of elements distribution in a specimen), the EDX analysis is a clear indication of the surface degradation due to oxide layer formation covering the synthesized alloys. The oxidation is inevitable in ferromagnetic nanoparticles, and they can influence the magnetic properties of the alloys in which Ni_2_O_3_ is weakly magnetic; NiO is antiferromagnetic, Ni(OH)_2_ is nonmagnetic whereas Fe_2_O_3_ is ferromagnetic [45].

**Figure 6:**
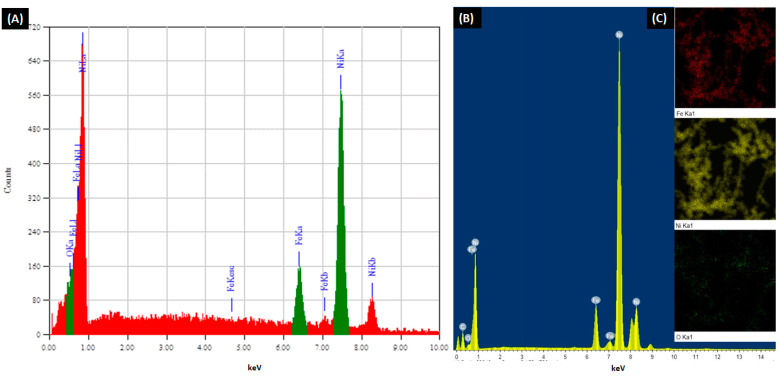
EDX analysis of the synthesized Fe_10_ Ni_90_ alloy using (**A**) SEM and (**B**) TEM. (**C**) TEM mapping of the alloy. Reaction reference: 0.1 M metal concentration, 80 mL reaction volume, 1400 rpm, and 95–98 °C.

As shown in Table 1, the alloys with Fe_10_ Ni_90_, Fe_20_ Ni_80_, and Fe_30_ Ni_70_ molar ratio possess high purity with minimal oxides forming, as confirmed by both TEM and SEM instruments with differences in the X-ray source, and its depth of penetration (i.e., In SEM, the source of X-rays is a sphere with a diameter of about 1000 nm, whereas, in TEM, the source of X-rays is a disk with beam’s diameter and a thin (50–100 nm) section). The increase in the iron content (molar ratio) to more than 30% resulted in an increase in the oxygen content of the synthesized alloy; for that, the alloy purity significantly decreased with a higher molar ratio than Fe_30_ Ni_70_ (especially alloy Fe_50_Ni_50_ molar ratio). Indeed, the Fe_10_Ni_90_ molar ratio alloy is very pure without oxides forms as supported by a full agreement between EDX analyses using both SEM and TEM with their difference the X-ray sources and penetration. The EDX analysis of the synthesized Fe_10_Ni_90_ alloy using both SEM and TEM, as well as the TEM mapping of its composition, shown in Figure 6.

**Figure 7:**
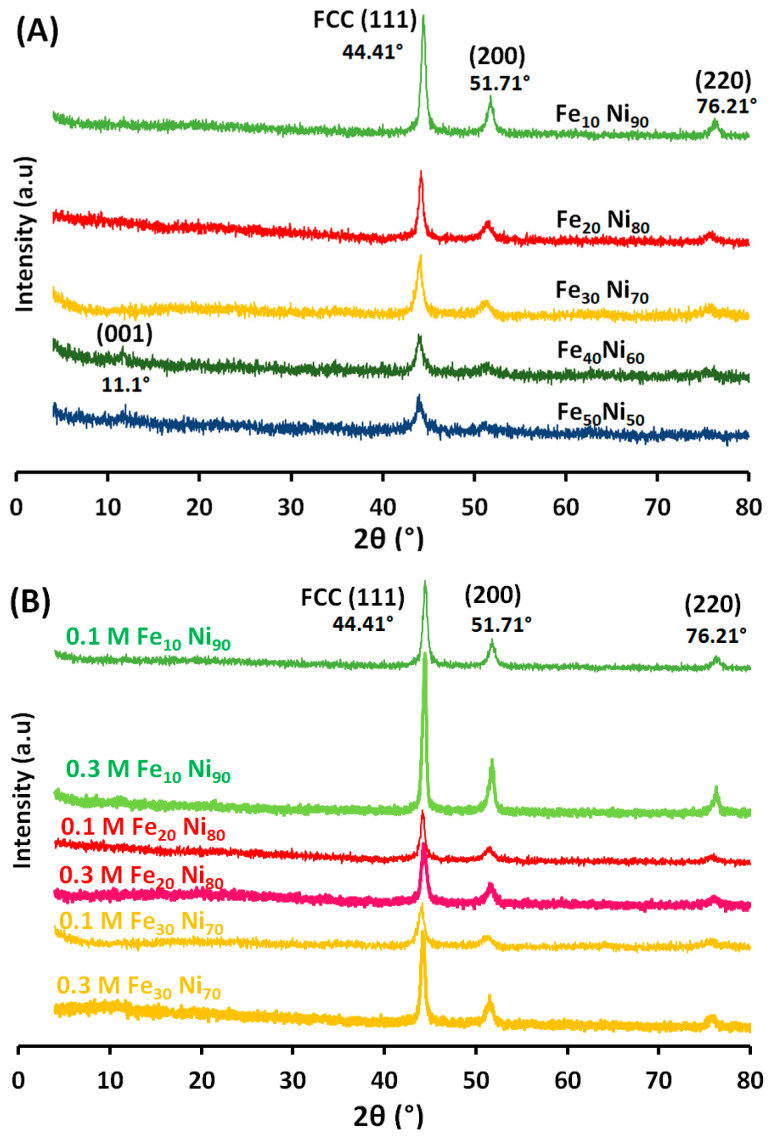
XRD pattern of the prepared iron-nickel alloys using (**A**) different iron-nickel molar ratio with 0.1 M metal concentration and 80 mL reaction volume, (**B**) 10 to 30% iron molar ratio at both 0.1 and 0.3 M metal concentration and 80 mL reaction volume. Reference condition: 1400 rpm, and 95–98 °C.

In full support of the obtained EDX results, the XRD pattern of the different alloys is shown in Figure 7. From the literature, the diffraction peaks correspond (111), (200), and (220) reflections of γ-nickel with face-centered cubic structure (fcc). The synthesized alloys in full agreement with the face-centered cubic (fcc) nickel crystal (JCPDS Card No. 04-0850), and the peaks located at 44.41°, 51.71°, and 76.21° can be indexed to (111), (200), and (220) planes of the crystalline face-centered cubic (fcc) FeNi_3_ alloy (JCPDS 65-3244). This proved that Ni^2+^ and Fe^2+^ salts were almost reduced to zerovalent metals and formed FeNi_3_ alloy. The strong and sharp peaks and very low backgrounds revealed that γ-Ni particles had a high degree of crystallization and no characteristic peaks due to impurities of nickel or iron oxides and hydroxides, which indicates pure alloy formation especially with the iron molar ratio 10, 20, and 30% (Fe_10_ Ni_90_, Fe_20_ Ni_80_, and Fe_30_ Ni_70_). The XRD peaks of hexagonal plates can be indexed as hexagonal Ni(OH)_2_ (JCPDS Card No. 14-0117) and fcc nickel. The broad Ni(OH)_2_ diffraction peaks such as (001) show a poorly crystallized layered phase with a turbostratic disorder for the presence of Cl^-^ in the case of nickel chloride, which was used as the nickel source [16], and appeared at an iron molar ratio higher than 30% as shown in Figure 7A for Fe_40_ Ni_60_ and Fe_50_ Ni_50_ molar ratios. The good purity of the three first iron molar ratios (Fe_10_ Ni_90_, Fe_20_ Ni_80_, and Fe_30_ Ni_70_) does not change with tripling the metal concentration as shown in Figure 7B. In other words; X-ray diffraction presented a complete phase purity for the alloys synthesized in this work using an iron molar ratio not exceeding 30%.

The average crystallite sizes of the iron-nickel alloys synthesized at various molar ratios were calculated based on the full width at half maximum of the (111) peak in the corresponding XRD patterns, using the Scherrer formula [46]. The data range from 15.88 to 7.93 nm corresponding to Fe_10_Ni_90_ to Fe_50_Ni_50_, respectively, indicating that all the synthesized iron-nickel alloys are nanostructured, regardless of the synthesis conditions.

**Figure 8:**
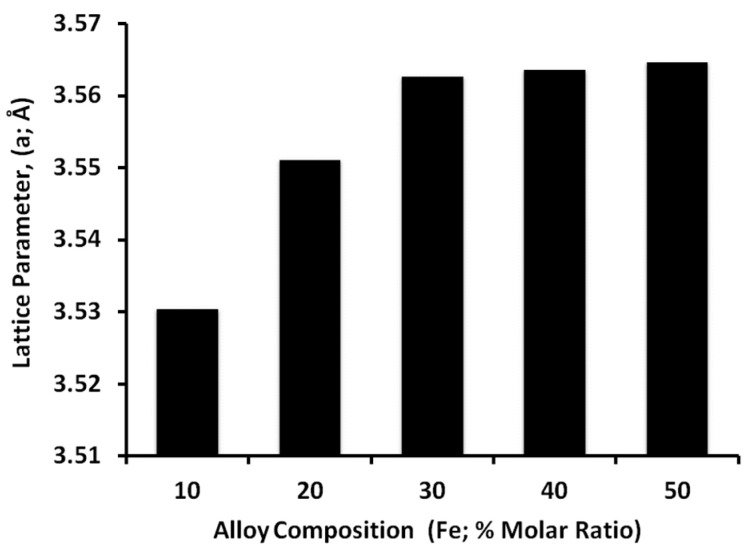
The lattice parameter as function of alloy composition (% Fe molar ratio).

As shown in Figure 8, the lattice parameter of the fcc FeNi alloys is filling between the lattice parameters of pure fcc nickel (3.52 Å) and pure fcc iron (3.59 Å). As noticed, the lattice parameter of the Fe_10_ Ni_90_ alloy is closer to that of pure nickel spectra as the prepared alloy is rich in nickel atoms. This can be related to the molar ratio of Fe^2+^ to Ni^2+^ that is equal or near to 1/3; Ni^2+^ and Fe^2+^ were completely reduced into Fe and Ni, resulting in the formation of (FCC) FeNi_3_ phase [47,48,49]. The increase in the iron molar ratio to Fe_20_ Ni_80_ and Fe_30_ Ni_70_ resulted in shifting the lattice parameter toward the pure iron that indicated iron consolidation into the lattice of nickel in which the ionic radius of iron is different than that of nickel.

Figure 8 does not agree with Vegard’s law [50] for iron molar ratio higher than 30%, according to which the crystal lattice parameter of the alloys should increase linearly with an increase in the concentration of larger iron atoms. A deviation from Vegard’s law occurs in case of x = 10–30, the solid solution is ordered to form the FeNi_3_ phase, whose parameter a = 3.55 Å (JCPDS Card No. 01-077-7971). An increase in the lattice parameter for the alloy at x > 30, and a slowdown in the growth for x = 40 or 50 may be due to the conditions of the stoichiometry of the superstructure phase of FeNi_3_ and the presence of defects in it, as well as the dimensional effects of nanocrystals.

### 3.4. Alloy Reaction Mechanism

The reaction progress in the current route could be expressed as the following equations:

2 N_2_H_4_ + 8 OH^−^ ↔ 2 N_2_ + 8 H_2_O + 8 e^−^ (1)

2 Fe^2+^ + N_2_H_4_ + 4 OH^−^ → 2 Fe + N_2_ + 4 H_2_O (2)

2 Ni^2+^ + N_2_H_4_ + 4 OH^−^→ 2 Ni + N_2_ + 4 H_2_O (3)

Fe^2+^ + Ni^2+^ + 4e^−^ → FeNi (4)

Fe + 3 Ni → FeNi_3_ (5)

When Fe(III) and Ni(II) are treated with alkaline hydrazine hydrate, it resulted in Fe(OH)_3_, which is easily formed. Ni(II) is readily reduced to Ni particles for cooperation with hydrazine hydrate, which in turn reduces Fe(OH)_3_ to Fe particles. In case of equal or near the 1:3 molar ratio of Fe^3+^ to Ni^2+^ ions, both Fe^3+^ and Ni^2+^ can be completely reduced into Fe and Ni, resulting in the formation of fcc FeNi_3_ phase with AuCu_3_ type structure. Based on a precipitate slow-release process, Fe^3+^ and Ni^2+^ can be slow-released from Fe(OH)_3_ and Ni(OH)_2_ by high-temperature decomposition, and consequently, the concentrations of Fe^3+^ and Ni^2+^ are kept at a 1:3 ratio level, which is in favor of anisotropic growth. Fractal growth is a nonequilibrium, diffusion-controlled kinetic process, which has been supported by the diffusion-limited aggregation (DLA) [51] or oriented aggregation mechanism [52].

### 3.5. Thermal Properties

**Figure 9:**
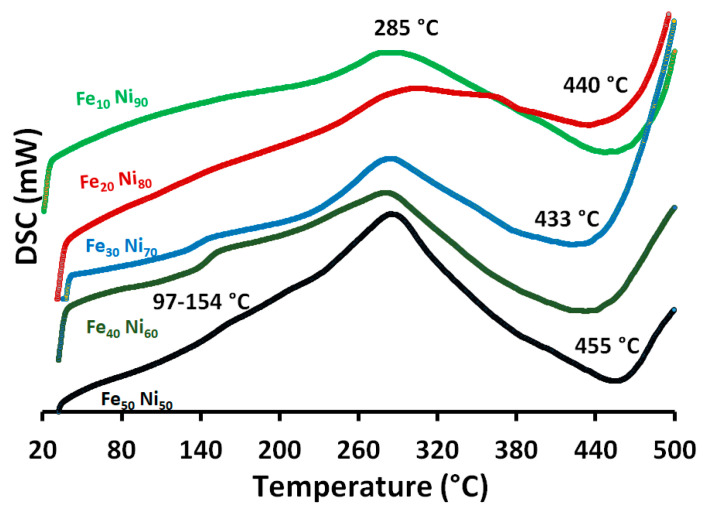
The DSC diagram of the prepared iron-nickel alloys using different iron-nickel molar ratio (10–50% Fe). Reaction reference: 0.1 M metal concentration, 80 mL reaction volume, 1400 rpm, and 95–98 °C.

In the DSC diagram shown in Figure 9, two endothermic peaks were identified. The first broad peak was around 97–154 °C and around 433–455 °C, respectively. The endothermic peak at 97–154 °C can be attributed to water evaporation. This peak is pronounced for alloys with Fe_30_ Ni_70_, Fe_40_ Ni_60_, and Fe_50_ Ni_50_ molar ratios. Clearly, this peak does not exist in Fe_10_ Ni_90_ and Fe_20_ Ni_80_ alloys. The second endothermic peak around 433–455 °C can be attributed to the Curie transition temperature (i.e., the Curie temperature is the temperature above which magnetic materials lose their ferromagnetic properties, to be replaced by paramagnetism). The Curie transition temperature of nanoparticles is lower than that of the bulk, which occurs at temperatures close to 580 °C [29]. The decrease in the particle size leads to a decrease in the overall ordering of the magnetic phase, which can be related to both fractions of the atoms on a disordered surface being less tightly subjected to a super-exchange interaction and less ordered crystal structure in comparison to the grain interior [47]. The broad endothermic peak occurs dependent on the crystallite sizes, where the transition takes place from the pc (i.e., The primitive cubic system (cP) consists of one lattice point on each corner of the cube). The (111) plane of a face-centered cubic system is a hexagonal grid. On the other hand, the remarkable exothermic peak at 285 °C was observed, which can be attributed to crystallization, in which a grain-size increase would accompany a crystallization process.

### 3.6. Magnetic Properties

**Figure 10:**
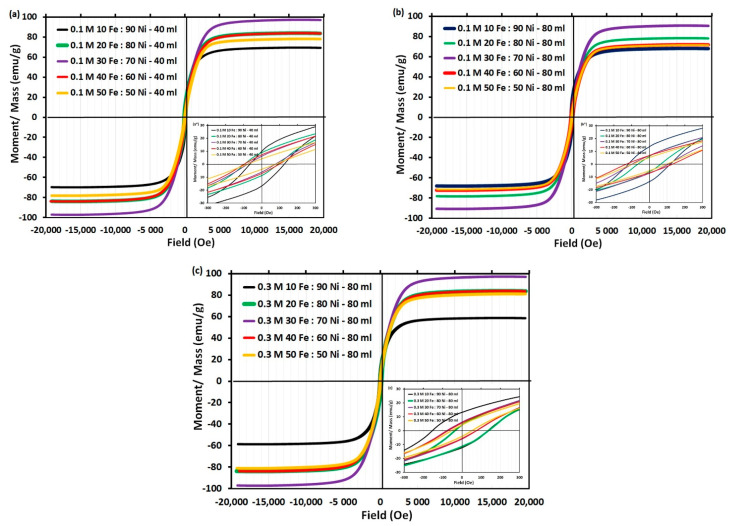
The M-H hysteresis loops of the iron-nickel microcrystals at room temperature; (**a**) 0.1 M metal concentration in 40 mL reaction volume, (**b**) 0.1 M metal concentration in 80 mL reaction volume, and (**c**) 0.3 M metal concentration in 80 mL reaction solution. Reaction reference: 1400 rpm and 95–98 °C.

Figure 10 shows the hysteresis loop of synthesized iron-nickel (FeNi_3_) alloy. It indicates that the sample has the symmetric hysteresis loop behavior of ferromagnetic materials. The presented iron–nickel in this work shows a wide range of superior magnetic properties that can be tailored to the need of certain requirements by adjusting the chemical composition. The highest coercivity without significant reduction in saturation magnetization reveals that the magnetic behavior of Fe_10_Ni_90_ alloy surpasses that of soft ones.

The larger coercivity of the FeNi_3_ starfish-like structure in Fe_10_ Ni_90_ alloy could be attributed to the intrinsic large magnetocrystalline anisotropy of ordered intermetallic compared with the disordered FeNi_3_ permalloy. Also, the effect of size, shape, and structure compared with the FeNi_3_ necklace-like or particles of other synthesized alloys. Indeed, other parameters such as composition, internal stress, and defects also influence alloy magnetic properties. The magnetic properties of FeNi_3_ structures may be explained depending on crystalline and shape anisotropy, which needs to be studied further.

**Figure 11:**
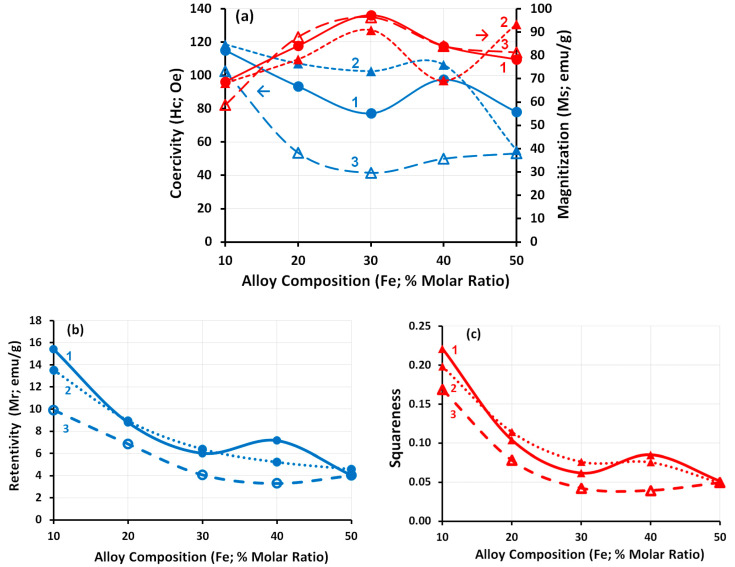
Magnetic Properties as function of Iron molar ratio in the alloys; (**a**) Coercivity and Magnetization, (**b**) Retentivity, and (**c**) Squareness. Reference conditions: Curve 1: 0.1 M metal concentration in 40 mL reaction volume, Curve 2: 0.1 M metal concentration in 80 mL reaction volume, and Curve 3: 0.3 M metal concentration in 80 mL reaction solution.

For a clear study of the magnetic properties; coercivity, magnetization, receptivity, and squareness were drawn as functions of the iron molar ratio, as shown in Figure 11. The Fe_10_ Ni_90_ alloy shows a high coercivity of 115.06 Oe in the case of 40 mL reaction volume, which improved to 118.55 Oe with doubling the reaction volume (80 mL). These values slightly decreased with increasing the iron content in the alloy up to Fe_30_ Ni_70_ alloy (77.24 and 102.53 Oe for 40 and 80 mL reaction volume, respectively).

Although we observed increases in the coercivity by doubling the reaction volume for all studied iron molar ratios except Fe_50_ Ni_50_ alloy, an increase in the metal concentration from 0.1 (Curves 1 and 2; Figure 11) to 0.3 M (Curve 3; Figure 11) resulted in a clear significant reduction in the coercivity of all synthesized alloys. However, Fe_10_ Ni_90_ alloy kept a minimum reduction in its coercivity by tripling the total metal concentration. Conversely, the same saturation magnetization (Ms) (68.7 and 68.2 emu g^−1^ for 40 and 80 mL reaction volume, respectively) is observed for the Fe_10_ Ni_90_ alloy. The Ms increased with an increase in the iron content in the alloy up to 30% (97.2 and 90.9 emu·g^−1^ for 40 and 80 mL resection volume, respectively). The increase in Ms with an increase in the iron content of the alloy is due to the high inherent magnetization of iron [48]. The increased magnetization could be established by the Slater–Pauling curve [49,53]. In full agreement with previous work [54], increasing iron concentration in the alloy results in an increase in Ms whereas Hc decreases proportionally. The highest Retentivity (Mr) and Squareness were recorded for Fe_10_ Ni_90_ alloy that decreased with increases the iron molar ratio. More pronounced is the nearly stable, high magnetic properties of the Fe_10_ Ni_90_ alloy with different reaction conditions (i.e., doubling the reaction volume and/or tripling the total metal concentration).

## 4. General Discussion

Although it is possible to prepare the nanostructured alloy from iron and nickel in different ways, most of these methods suffer from problems that may reduce the purity of the resulting alloy and affect its magnetic properties. These problems emerge due to the following reasons: (1) the use of an expensive equipment such as autoclave, or special preparation capabilities and conditions such as the reflux process, (2) the use of auxiliaries as surfactants, templates or organic solvents, whether for preparation or washing, which negatively affect the purity of the alloy and increase its manufacture costs, (3) the preparation time is long, up to 10 h, in addition to the drying time, (4) the magnetic properties are low to medium in relation to the cost, conditions and quality of the devices used for preparation, (5) the scarcity of the alloy composition of iron and nickel rich in nickel (Fe_10_ Ni_90_) and the low purity of the prepared alloys, (6) there are no sufficient studies to prepare high magnetic properties of Fe_10_ Ni_90_ using simple, inexpensive and easy-to-control methods such as simple chemical reduction, and (7) there are no studies documenting the study of the effect of the volume of the reaction solution on the purity and properties of iron and nickel alloys. All these defects have been overcome in this study.

Generally, the preferential growth direction of alloy particles follows the route of the minimum magnetic anisotropic energy and/or surface free energy. The ferromagnetic iron-nickel alloy has an fcc structure that possesses a lower surface energy value than (100) and (110). For that, the magnetic particles grow along the magnetic easy axis of (111) direction, and thus (111) consists of more facets. On other hand, fast grain growth leads to a similar growth rate of various facets and isotropic morphology, such as spheres, which are usually formed. Whereas, at low grain growth rate, the alloy particles have enough time to grow along the preferential direction. Hence, an anisotropic growth along the (111) direction occurs, driven by the minimization of magnetic anisotropic energy and surface free energy. From this point, the OH^−^ ions play an important role in the reduction and particle growth in which it complexes the metal ions and acts as a capping agent. There are three cases depending on the molar ratio of free OH^−^/M^+^ ions; the first case is if the molar ratio of free OH^−^/M^+^ ions is high, in which OH^−^ ions are enough to completely cap the growing particles. The opposite case is if the molar ratio of free OH^−^/M^+^ ions is low in which OH^−^ ions are not enough to cap any face of the growing particles. The previous two cases lead to isotropic particle growth. The third case is the medium molar ratio of free OH^−^/M^+^ ions in which partially cap the growing particles and anisotropic particle growth along the preferential direction takes place [31]. However, in this work, the ratio of free OH^−^/(Fe^2+^ and Ni^2+^) ions should not change as the reaction goes on because both types of ions are consumed according to the stoichiometric ratio. Accordingly, the effect of the ratio between Fe^2+^ and Ni^2+^ ions in the reaction medium is highlighted. By beginning with molar ratio Fe_10_ Ni_90_, and the presence of excess nickel ions and hydrazine, and because the bond energy between OH^−^ and nickel ions is larger than that between hydrazine and nickel ions, then it is apparent that the chemical bond between nickel ions and hydrazine is more likely to form before the reduction, which influences the growth rate of some crystal faces that result in the anisotropic growth; finally, the starfish-like shape morphology is formed. Moreover, the atomic percentage of oxygen in Fe_10_ Ni_90_ alloy is up to 0.05%, indicating that no oxide layer formed on the particle surface.

The strong and sharp peaks revealed that γ-Ni particles had a high degree of crystallization. The FeNi_3_ starfish-like nanostructured alloy is more stable; this proves that iron and nickel ions in the reaction solution are almost reduced to zerovalent metals and secure from further oxidation. With increasing the iron ions and decreases the nickel ions, a very slight shift in the XRD sharp single phase peak position is observed, indicating iron consolidation into the lattice of nickel due to the different ionic radius of iron. This implies that Fe atoms are incorporated into the fcc Ni lattice, leading to the enlargement of the interplanar spacing because the radius of Fe atoms (1.26 Å) is larger than that of Ni atoms (1.24 Å).

On the other hand, the lower possibility of bonding nickel and OH^−^ ions makes the argument for strengthening OH^−^ ions’ role as capping agent and the growth of the three crystal planes (100, 110 and 111) with an isotropic particle growth. The necklace-like nanostructured consists of several nanospheres, which are collected together and form the necklace-like shape that can be illustrated by a dipole-directed assembly mechanism [22]. All dipoles could be located in a group of nanospheres, which resulted in a decrease in the magnetostatic energy and in the spontaneous magnetostatic field, and consequently resulted in the degradation of dipoles in the manner of interaction between the unpaired end dipoles of two or several nanoparticles/chains. Necklace-like nanostructured chains with the same magnetostatic direction are linked together by a second magnetostatic field that would induce self-assembly, and a necklace-like is formed [55]. Further increases of the iron ions relative to nickel ions, push the isotropic particle growth with the appearance of oxides layers with an iron molar ratio higher than 30% in which α-Fe particles with low nickel content could be oxidized as confirmed by XRD. Conversely, iron can be oxidized faster than nickel; a faster growth leads to a similar growth rate of various facets and isotropic spheres, which was obtained. Also, a deviation from Vegard’s law was recorded for an iron molar ratio higher than 30%, in which superstructure phase of FeNi_3_ was formed and the presence of defects in it, as well as the dimensional effects of nanocrystals [56].

The role of metal concentration in controlling the morphology of the alloy particles can be attributed to (1) the generation rate of iron and nickel atoms and therefore the growth rate of alloy particles and (2) the molar ratio of free OH^−^/(Fe^2+^ and Ni^2+^) ions in the reaction solution. An increase in iron ion concentration in the alloys results in an increasing trend in Ms, while Hc decreases proportionally.

This work provides a simple, effective, and low-cost synthetic method to prepare stoichiometric FeNi_3_ alloy.

## 5. Conclusions

Nanostructured magnetic materials are highlighted due to their unique properties such as superparamagnetic, high magnetic anisotropy and coercivity, and giant magnetoresistance. These phenomena arise from finite size and surface effects in which the microstructure of materials has much influence on their physical and chemical properties. Nanocrystalline iron-nickel alloy receives attention because of its remarkable ferromagnetic properties and unique mechanical characteristics. Although it is possible to prepare the nanostructured alloy from iron and nickel in different ways, most of these methods suffer from problems that may reduce the purity of the resulting alloy and affect its magnetic properties.

In this work, iron-nickel nanostructure alloys with particle sizes in the range 77–532 nm have been successfully prepared via simple chemical reduction, which is characterized by simplicity, low cost, and easy control of the preparation conditions. In addition, no surfactants, templates, or organic solvents were used either for the preparation or washing process.

The nanostructured Fe_10_ Ni_90_ alloy, which is characterized by the distinctive sea starfish-like shape with high magnetic and unique morphological properties and with a reaction time not exceeding 15 min, was investigated. The length and width of the cones/needles increase in diminutions with an increase in the volume of the reaction mixture or the concentration of the metal. The prepared alloy is also characterized by high purity (99.9%) as well as high stability due to the low concentration of rapidly oxidizing iron in the mixture, which gives it superior magnetic properties that enables it to be used in multiple applications such as medical and environmental applications such as removing water pollutants, and separating gases, etc.

The morphology of the synthesized alloy significantly changed with the initial molar ratio of metal ions. A deviation from Vegard’s law was recorded for an iron molar ratio higher than 30%, in which the superstructure phase of FeNi_3_ was formed and the presence of defects in it, as well as the dimensional effects of nanocrystals.

A remarkable change (improvement) in the magnetic properties of the alloy with an increase in the volume of the reaction solution was recorded. Increases in the total metal concentration decrease the coercivity (Hc) of the synthesized alloy. The prepared nanostructures and magnetic properties have many potential applications in nanoscience and material fields due to their scientific value.

## Figures and Tables

**Table 1:** EDX analysis of the synthesized five alloys equipped with TEM and SEM. Reaction reference: 0.1 M metal concentration, 80 mL reaction volume, 1400 rpm, and 95–98 °C.

Iron: Nickel Molar Ratio	TEM EDX Analysis						SEM EDX Analysis					
	Atomic %			Mass %			Atomic %			Mass %		
	Fe	Ni	O	Fe	Ni	O	Fe	Ni	O	Fe	Ni	O
10:90	11.36	88.64	0.00	10.87	89.13	0.00	10.74	89.22	0.05	10.27	89.72	0.01
20:80	17.50	71.71	10.78	18.24	78.54	3.22	20.05	79.05	0.89	19.39	80.36	0.25
30:70	28.51	62.44	9.06	29.47	67.85	2.68	31.25	67.22	1.53	30.53	69.04	0.43
40:60	34.88	47.76	17.36	38.73	55.75	5.52	39.89	56.60	3.51	39.20	58.27	2.53
50:50	39.06	40.19	20.74	44.77	48.42	6.81	41.92	42.82	15.26	45.91	49.30	4.79

## Data Availability

All data generated or analyzed during this study are included in this published article.
